# Evaluation of Foveal and Parafoveal Microvascular Changes Using Optical Coherence Tomography Angiography in Type 2 Diabetes Patients without Clinical Diabetic Retinopathy in South Korea

**DOI:** 10.1155/2020/6210865

**Published:** 2020-08-06

**Authors:** Young Gun Park, Minhee Kim, Young Jung Roh

**Affiliations:** ^1^Department of Ophthalmology and Visual Science, Seoul St. Mary's Hospital, College of Medicine, The Catholic University of Korea, 222 Banpo-daero, Seochu-gu, Seoul 06591, Republic of Korea; ^2^Department of Ophthalmology and Visual Science, Yeouido St. Mary's Hospital, College of Medicine, The Catholic University of Korea, 63-ro 10, Yeoungdeongpo-gu, Seoul 07345, Republic of Korea

## Abstract

The aim of this study was to investigate foveal and parafoveal microvascular changes in retinal vascular plexuses in patients with type 2 diabetes mellitus (DM) without clinical diabetic retinopathy (NDR) using optical coherence tomography angiography (OCTA) in South Korea. We included 64 patients in the NDR group and included 48 healthy control subjects for comparison. All subjects underwent ocular examination with visual acuity and wide-field fundus photos. Foveal and parafoveal vessel density and foveal avascular zone (FAZ) area (mm^2^) in the superficial capillary plexus (SCP) and deep capillary plexus (DCP) were analyzed. Foveal vessel densities in both the SCP and DCP were decreased in the NDR group compared to the controls (*p* = 0.034 and 0.001, respectively). Vessel densities in the superior and inferior parafoveae in the DCP were decreased in the NDR group compared to the controls (*p* = 0.006 and 0.034, respectively). The FAZs of the SCP and DCP were significantly different between the NDR group and the controls (*p* = 0.003 and 0.001, respectively). The average vessel densities of the SCP and DCP were not correlated with HbA1c, serum creatinine, or the duration of DM in the NDR group. We demonstrated that OCTA can identify early-stage DR before the manifestation of clinically apparent retinopathy in diabetic eyes. Diabetic patients without clinical DR have microvascular alterations (foveal vessel density, parts of the parafovea, and enlarged FAZ) in the SCP and DCP. Our results suggest that OCTA might be a promising tool for early detection of eyes with DR.

## 1. Introduction

Diabetic retinopathy (DR) is one of several complications of diabetic mellitus (DM) and an important cause of blindness worldwide [1, 2]. Efforts in early detection and screening for DR could reduce the severity and possibility of blindness. It is difficult to reverse the damage from DR, and the risk of DR progression increases once retinal lesions become clinically visible [3]. Having a clearer understanding of the early changes in DR might provide novel and more effective preventive strategies. Thus, it is necessary to detect and monitor the subtle microvascular changes in patients with DM and subclinical DR.

The most common early clinically visible manifestations of DR include microaneurysm formation and intraretinal hemorrhage. At present, ophthalmoscopy and color fundus photography are still the gold standards for the diagnosis and staging of DR [4, 5]. However, microvascular damage is known to occur before findings of retinopathy become apparent on clinical examination or fundus photography. Although fluorescein angiography (FA) is an important modality in revealing capillary leakage and nonperfusion in patients with DM, it is rarely used and not suggested for eyes without visible retinopathy or mild DR.

Optical coherence tomography angiography (OCTA) is a novel and noninvasive angiographic technique without intravenous dye injections. It can easily show vessel densities, vascular abnormalities in retinal blood flow, and the shape of the foveal avascular zone (FAZ) [6]. The microvascular dropout of the macula in early-stage DR has been reported in recent OCTA studies [7, 8]. Recently, advances in projection artifact removal allowed to accurately define not only the superficial plexus but also the deep retinal vascular layers. Some researchers reported microvascular changes in the SCP and DCP in patients with diabetes without retinopathy [9, 10]. However, there has not been much research in quantitative microvascular changes on the different vascular layers of the retina using OCTA in patients with preclinical DR. The aim of this study was to investigate retinal microvascular alterations of the foveal and parafoveal areas and compare these changes between diabetic eyes with no signs of DR and healthy normal controls using OCTA in South Korea.

## 2. Materials and Methods

### 2.1. Subjects

This study was a retrospective review of consecutive cases. Patients attending the Department of Ophthalmology of Seoul St. Mary's Hospital in Seoul, Korea, between January 2018 and January 2019 with a confirmed diagnosis of type 2 DM without clinical DR were included. The healthy control group included healthy patients attending medical checkups with no posterior segment abnormalities or systemic comorbidities. All procedures were conducted in accordance with the Declaration of Helsinki and its later amendments. The study was approved by the ethics committee of Seoul St. Mary's Hospital and the Catholic University of Korea. The need to obtain informed patient consent was waived due to the retrospective design of the study.

All patients and controls had their best-corrected visual acuity (BCVA) measured initially and then underwent standardized dilated fundus examinations, including measurements by swept-source OCT (SS-OCT) and OCTA imaging (DRI OCT Triton; Topcon, Tokyo, Japan). Subjects underwent fundus examinations and ultrawide-field fundus photography capable of capturing 200° of the retina (Optos California; Dunfermline, United Kingdom). After that, two experienced examiners (YGP and MHK) reviewed the fundus photographs and confirmed eyes with no diabetic retinopathy (NDR) [11]. In both patients and controls, the exclusion criteria were any other ocular disease that may affect ocular circulation (e.g., glaucoma, age-related macular degeneration, and refractive error > 5 diopters), intraocular surgery, or severe media opacity (e.g., lens opacity due to cataract or thick asteroid hyalosis).

### 2.2. Optical Coherence Tomography Angiography

**Figure 1:**
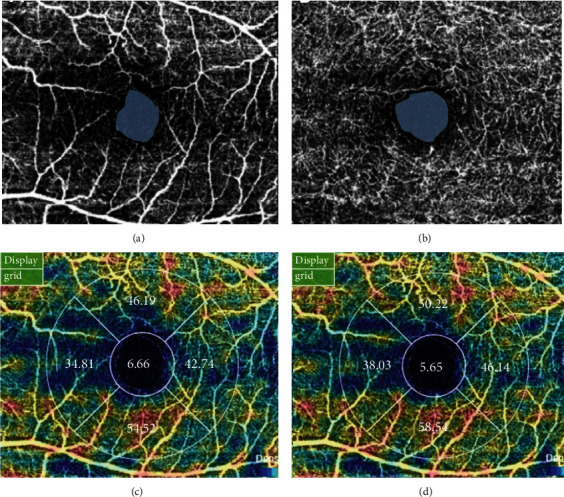
(a, b) Measurements of foveal avascular zone area and (c, d) results of vessel density measurements using optical coherence tomography angiography in a diabetic eye without clinical diabetic retinopathy (superficial capillary plexus, deep capillary plexus).

OCTA was performed with a DRI OCT Triton (Topcon). This instrument has an A-scan rate of 70,000 scans/s with an 840 nm wavelength light source and a 45 nm bandwidth. Patients with low-quality images (signal strength index < 50) were excluded. OCTA images were evaluated using automatic segmentation. We analyzed the vascular density of the superficial and deep retinal vascular zone within 1 and 3 mm inner and outer circles using computer software. The extent of the FAZ was also manually measured on the OCTA images of each participant by two investigators (YGP and MHK) who were blinded to each other (Figure 1).

### 2.3. Statistical Analysis

Unpaired *t*-tests were used to compare groups, and Pearson's correlation coefficients were used for correlation analysis. *p* < 0.05 was considered statistically significant. All analyses were produced using commercial software (SPSS version 22.0; IBM, Armonk, NY).

## 3. Results and Discussion

Sixty-four NDR eyes and forty-eight age-matched control subjects were included. Baseline demographics were comparable between the two cohorts (Table 1). The mean age of patients with DM was 61.0 ± 9.34 years while that of the controls was 62.94 ± 11.2 years (*p* = 0.729). DM patients had an average HbA1c of 7.48 ± 1.28%, average serum creatinine of 0.92 ± 0.31 mg/dL, and e-GFR of 82.67 ± 21.72 mmol/L. The average duration of diabetes for DM patients was 7.85 ± 4.37 years (Table 1).

The foveal vascular densities of both the SCP and DCP were significantly lower in the NDR group than in the control group (13.81 ± 4.37% vs. 15.77 ± 3.45%, 11.53 ± 4.41% vs. 13.68 ± 3.59%; *p* = 0.034 and *p* = 0.001, respectively). The parafoveal vascular density in the SCP did not differ significantly between the two groups (superior/temporal/inferior/nasal; all *p* > 0.05). Although the average parafoveal vessel densities in both the SCP and DCP were decreased in eyes with DM, only the vessel densities of the superior and inferior parafoveal areas in the DCP were significantly decreased in the NDR group compared to those in the controls (superior/temporal/inferior/nasal: *p* = 0.006, *p* = 0.395, *p* = 0.034, and *p* = 0.079, respectively; Table 2).

The correlation between the average vessel density of the SCP, the DCP, and HbA1c was unremarkable (*p* = 0.264 and 0.19, respectively). The average vessel densities of the SCP and DCP were not correlated with serum creatinine (*p* = 0.424 and 0.395, respectively). No significant correlation was found between the duration of diabetes and vessel density in the SCP or DCP, either (*p* = 0.648 and 0.713, respectively).

Reduction of the DCP network in the superior and inferior parafoveae and foveae was identified in the NDR group. Only the foveal vascular density in the SCP was lower in the NDR group than in the control group. This suggests a relationship between disease progression and vascular changes.

**Figure 2:**
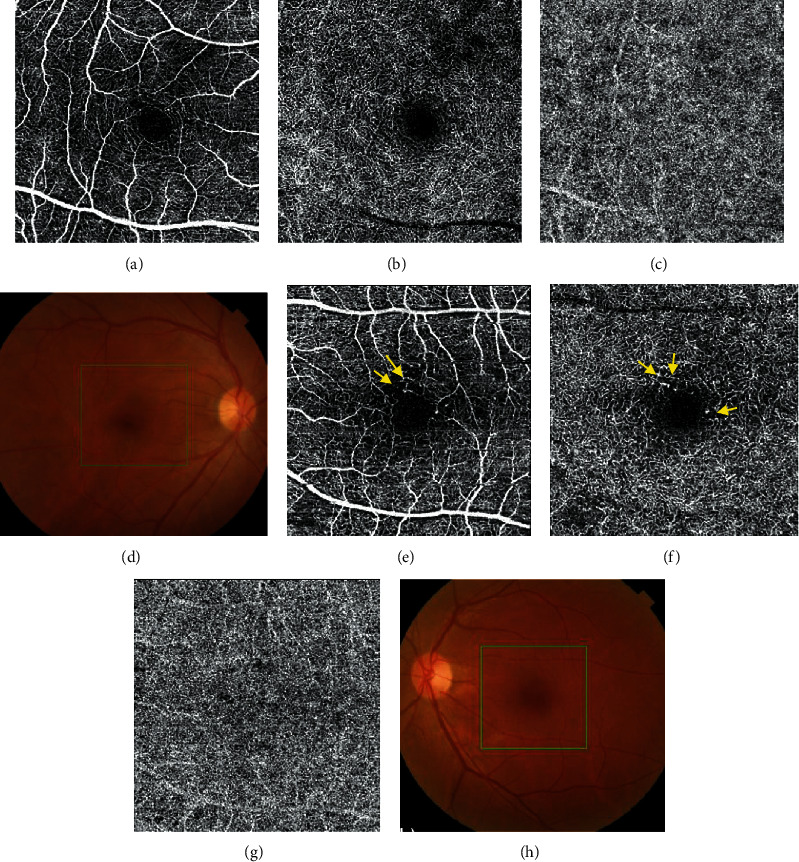
Optical coherence tomography angiography of a control eye (a–d) and a diabetic eye without clinical diabetic retinopathy (e–h) (superficial capillary plexus, deep capillary plexus, choriocapillary, and fundus photography from left to right). A few capillary nonperfusion areas with microaneurysm were observed in the diabetic eye (arrows).

The FAZs of the SCP and DCP were significantly enlarged in the NDR group when compared with those in the control group (0.34 ± 0.11 mm^2^ vs. 0.31 ± 0.08 mm^2^, *p* = 0.003; 0.55 ± 0.13 mm^2^ vs. 0.43 ± 0.07 mm^2^, *p* = 0.001, respectively; Table 2). In addition, microaneurysms were found in six diabetic eyes (9.38%) (Figure 2). We observed that the vessel densities of the SCP and DCP were not significantly associated with HbA1c, serum creatinine, e-GFR, or duration of diabetes. There was also no significant correlation between BCVA (logMAR), FAZs, and vascular density in the SCP or DCP in the NPR group.

## 4. Discussion

OCTA can selectively visualize specific retinal layers, which can be used to compare microvascular changes in patients without detectable clinical retinopathy [12, 13]. There is evidence that OCTA can identify subclinical microvascular alteration in various diseases. In particular, it could more accurately diagnose early-stage DR [14, 15]. Previous studies have reported changes in FAZs or macular vessel density in DR but with different results [16, 17].

Our study compared superficial and deep retinal vascular density and FAZs in type 2 DM patients with NDR and in control subjects by OCTA and assessed the correlation of earlier diabetic changes. As we obtained foveal and parafoveal vessel density within circles 1 and 3 mm in diameter, changes in foveal and parafoveal lesions could be separated and compared. We demonstrated that significant foveal vascular density changes occur in the SCP and DCP layers in NDR groups compared to healthy controls, and the changes in parafoveal vascular density only appeared in superior and inferior DCP lesions. There were no significant differences in the parafoveal vascular density of the SCP between groups.

These results may be due to small differences in vascular density in early-stage DM patients, which were not reflected over the whole macula. However, it implies that subtle microvascular changes have already started in diabetic retinas even in the absence of visible retinal lesions. In our study, there was only a significant decrease in vessel density in the superior and inferior parafoveal areas in the DCP layer. Unfortunately, the reason why this part was vulnerable is not clear and more research will be needed.

Changes in FAZs have recently been reported after the introduction of OCTA, but previous studies have provided different conclusions about this in diabetic patients without DR [18]. Cao et al. found no significant difference in the FAZ of SCPs between two groups (0.32 ± 0.18 mm^2^ vs. 0.35 ± 0.09 mm^2^) [19]. On the other hand, de Carlo et al. reported an enlarged FAZ in the SCPs of diabetic eyes compared to control eyes (0.348 mm^2^ vs. 0.288 mm^2^, *p* = 0.04) [20]. Our study also showed significant differences in the FAZs of both the SCP and DCP layers between the NDR group and controls (0.33 ± 0.07 mm^2^ vs. 0.26 ± 0.07 mm^2^, 0.51 ± 0.13 mm^2^ vs. 0.38 ± 0.12 mm^2^, respectively; all *p* = 0.002). FAZs can vary between individuals, and so direct comparison may be difficult [21, 22]. However, alterations of FAZs may be an important marker to identify early-stage DR, which might be one of the earliest signs of diabetic damage to retinal vasculature.

The pathogenesis of DR is not completely understood. Microvascular changes in the retina due to retinal ischemia may occur from damage to the endothelial cells and pericyte loss in the retinal capillaries as the disease progresses [23, 24]. Some researchers reported that the DCP might be more vulnerable to ischemia than the SCP due to anatomical differences [25, 26]. The DCP drains into the superficial venules through vertical anastomoses, whereas the SCP is organized with transverse capillaries directly connected to the retinal arterioles with higher perfusion pressure [27]. This might be helpful in explaining our results.

The most common early visible manifestations of DR are microaneurysms and intraretinal hemorrhage with fundus examinations. However, microvascular changes in the macula are not easily identifiable and can be easily missed. Previous studies have demonstrated subtle changes in diabetic eyes without clinical manifestations [28, 29]. Some researchers have showed changes in retinal blood circulation with fluorescence angiography in DM patients without DR [30, 31]. A number of OCTA-related studies are currently underway [28, 32–34].

OCTA is a method that allows for a detailed representation of retinal microvasculature through the segmentation of individual retinal vascular layers, and it has been widely applied in the identification of various retinopathies [35, 36]. In our study, patients with type 2 DM without NDR also showed prominent changes in foveal vessel density in the SCP and, to some extent, parafoveal vessel density in the DCP compared to healthy controls. Altered vessel density at the DCP may imply a preference for the retinal vascular system in the pathogenesis of DR, as retinal venules originate from the deep retinal vascular layers. Sambhav et al. showed progressive vessel density reduction in the deep vascular layer from 25.23% in mild DR to 11.16% in severe DR [37]. The earlier alteration in the deep vascular layer probably reflects retinal venular widening, damage to capillary endings, and microaneurysms. It may also influence the breakdown of the blood-retinal barrier and the progression of DR.

Recently, Zeng et al. reported that peripapillary retinal nerve fiber layer thickness and radial peripapillary capillary density were significantly lower in their NDR group compared to healthy controls [38]. This also demonstrated that subtle retinal microvascular changes have already started, which might be associated with early functional changes. In addition, Sousa et al. detected the retinal vascular responses, a vasodilatory response and a vasoconstriction response, with OCTA [39]. They suggested that OCTA could provide a new way of studying retinal vascular changes before clinically detectable disease. Further studies are needed to clarify this in NDR patients.

There were a few limitations of our study. First, this was a cross-sectional observational study. A longitudinal study should be performed to explain the relationship between the microvascular changes of parafoveal lesions and disease progression in early-stage DR. Secondly, the sample size was relatively small and not large enough to draw clear conclusions. Thirdly, precise fundus examinations such as fluorescence fundus angiography were not available in early-stage DM patients without clinical DR. Lastly, OCTA image artifacts can interfere with accurate assessment of the actual status of the retinal microvasculature; for example, projection artifacts might interrupt visualization of the deep layer.

## 5. Conclusions

Our results suggested that microvascular alterations detected by OCTA in early-stage DM patients without DR can provide appropriate diagnosis and proper managements for patients with a risk of further clinically apparent DR in South Korea. If we could detect microvascular changes in early-stage DR, we could use a more cautious approach with regard to individual disease statuses. OCTA may be able to detect diabetic eyes at risk of developing retinopathy and to screen for diabetes quickly and noninvasively before a systemic diagnosis is made.

## Figures and Tables

**Table 1:** Baseline characteristics.

	NDR group	Control group	*p* value
Patients	64	48	
Mean age (years)	61.0 ± 9.34	62.9 ± 11.2	0.73
Male : female	36 : 28	26 : 22	0.45
HbA1c (%)	7.48 ± 1.28	N/A	
DM duration (years)	7.85 ± 4.37	N/A	
Serum creatinine (mg/dL)	0.92 ± 0.31	N/A	
e-GFR (mmol/L)	82.67 ± 21.72	N/A	
BCVA (logMAR)	0.07 ± 0.08	0.07 ± 0.09	0.62
Signal strength index	64.71 ± 7.34	67.86 ± 6.32	0.15

NDR: patients with type 2 diabetes mellitus without clinical diabetic retinopathy; N/A: not applicable; e-GFR: estimated glomerular filtration rate.

**Table 2:** Foveal and parafoveal vascular densities of the superficial capillary plexus and deep capillary plexus evaluated by optical coherence tomography angiography.

	NDR group	Control group	*p* value
Superficial capillary plexus			
Fovea (%)	13.81 ± 4.37	15.77 ± 3.45	0.034^∗^
Superior (%)	46.37 ± 5.17	48.82 ± 4.57	0.294
Temporal (%)	44.26 ± 4.75	45.68 ± 3.29	0.424
Inferior (%)	48.17 ± 6.74	47.38 ± 5.59	0.421
Nasal (%)	41.18 ± 4.34	43.27 ± 3.83	0.311
FAZ area (mm^2^)	0.34 ± 0.11	0.31 ± 0.08	0.003^∗^
Deep capillary plexus			
Fovea (%)	11.53 ± 4.41	13.68 ± 3.59	0.001^∗^
Superior (%)	48.28 ± 6.58	51.92 ± 4.67	0.006^∗^
Temporal (%)	47.35 ± 4.65	47.75 ± 3.97	0.395
Inferior (%)	47.36 ± 7.35	50.31 ± 6.39	0.034^∗^
Nasal (%)	44.34 ± 4.51	46.52 ± 4.01	0.079
FAZ area (mm^2^)	0.55 ± 0.13	0.43 ± 0.07	0.001^∗^

NDR: patients with type 2 diabetes mellitus without clinical diabetic retinopathy; FAZ: foveal avascular zone.

## Data Availability

The data used to support the findings of this study are available from the corresponding author upon request.
